# Bacterial Expression of Mouse Argonaute 2 for Functional and Mutational Studies

**DOI:** 10.3390/ijms11020745

**Published:** 2010-02-12

**Authors:** Vincenzo Salvatore, Nicoletta Potenza, Umberto Papa, Valentina Nobile, Aniello Russo

**Affiliations:** Department of Life Sciences, Second University of Naples, Via Vivaldi 43, 81100 Caserta, Italy; E-Mails: salvatore.enzo@gmail.com (V.S.); nicoletta.potenza@unina2.it (N.P.); umberto.papa@unina2.it (U.P.); valnob@tin.it (V.N.)

**Keywords:** RNA interference, microRNA, small interfering RNA, RNA-induced silencing complex

## Abstract

RNA interference (RNAi) is a post-transcriptional gene-silencing process that occurs in many eukaryotic organisms upon intracellular exposure to double-stranded RNA. Argonaute 2 (Ago2) protein is the catalytic engine of mammalian RNAi. It contains a PIWI domain that is structurally related to RNases H and possibly shares with them a two-metal-ion catalysis mechanism. Here we describe the expression in *E. coli* of mouse Ago2 and testing of its enzymatic activity in a RISC assay, *i.e.*, for the ability to cleave a target RNA in a single position specified by a complementary small interfering RNA (siRNA). The results show that the enzyme can load the siRNA and cleave the complementary RNA in absence of other cellular factors, as described for human Ago2. It was also found that mutation of Arg669, a residue previously proposed to be involved in substrate and/or B metal ion binding, doesn’t affect the enzymatic activity, suggesting that this residue doesn’t belong to the active site.

## 1. Introduction

RNA interference (RNAi) is a post-transcriptional gene-silencing process that occurs in many eukaryotic organisms upon intracellular exposure to double-stranded RNA (dsRNA) [1]. A ribonuclease (RNase) III family enzyme, Dicer, initiates silencing by processing dsRNA into ∼20 bp duplexes with two-nucleotide 3′ overhangs at both ends, called small interfering RNAs (siRNAs) [2–5]. The two strands of the siRNAs are then separated, and the one with the least stable 5′-end (the guide strand) is preferentially incorporated into the RNA-induced silencing complex (RISC), a ribonucleoprotein that uses its RNA component as a guide to endonucleolytically cleave complementary mRNAs [6–8]. RISC also mediates the function of endogenous, non-coding regulatory RNAs called microRNAs (miRNAs) [9,10].

The RISC component endowed with ribonucleolytic activity is Argonaute 2 (Ago2), a protein characterized by PAZ and PIWI domains, with the latter comprising the catalytic center [11,12]. The crystal structure of full-length Argonaute from *Pyrococcus furiosus* (PfAgo) has shown that the PIWI domain has an RNase H fold with two catalytic aspartates in positions identical to those found in other RNase H family enzymes [13]. These residues and an adjacent histidine bind a divalent metal ion (Mg^2+^ or Mn^2+^) and play a crucial role in catalysis [14]. Additional insights into the catalytic mechanism of Argonaute proteins have been provided by the determination of the crystal structure of *Bacillus halodurans* RNase H (Bh-RNase H) in complex with an RNA:DNA hybrid [15]. This study revealed that substrate binding is mediated by two Mg^2+^ ions. The first one (metal ion A) is equivalent to the metal ion observed in the crystal structure of PfAgo and is required for substrate-assisted nucleophile formation. The second magnesium ion (metal ion B), which is expected to stabilize the pentacovalent reaction intermediate, was found to be coordinated by the same two aspartates that bind metal ion A, a glutamate, and two oxygen atoms of the scissile phosphate. It was then proposed that metal ion B, not observed in the crystal structure of PfAgo, may bind to the active site of Argonautes upon interaction with the substrate [15].

Heterologous cell systems for protein expression and site-directed mutagenesis are very useful tools to inquire the mechanism of enzymatic reaction. Despite the great interest for Argonaute proteins and RNA interference, only two mammalian Argonautes, human Ago1 and Ago2 have been expressed and subjected to mutational studies [12,14,16]. Here we report the development of an expression system in *Escherichia coli* for mouse Argonaute 2 (mAgo2) that is suitable for functional and mutational studies.

## 2. Results and Discussion

### 2.1. Expression in E. Coli and Purification of Mouse Argonaute 2

**Figure 1.**
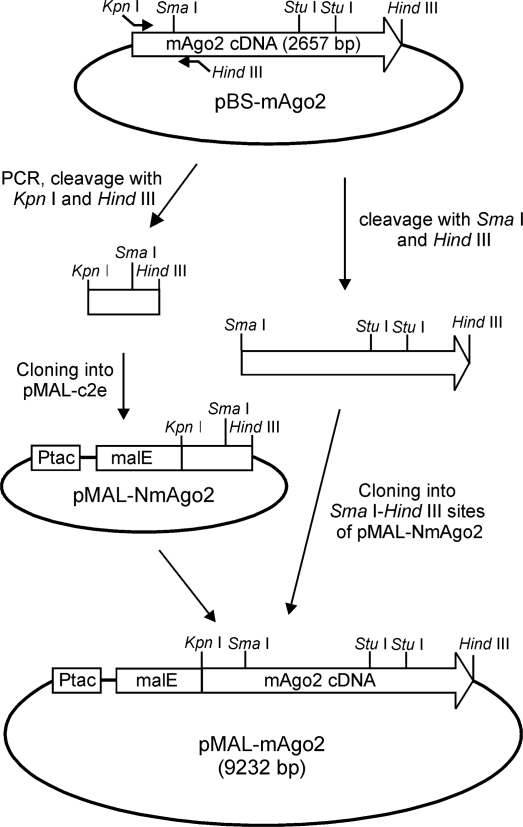
Construction of the expression vector pMAL-mAgo2. The cDNA coding for mouse Ago2 was cloned in frame with the 3′ end of the DNA encoding the maltose binding protein (MBP), under the transcriptional control of P_tac_ promoter. For description see the text.

In order to express in *E. coli* mAgo2, its coding sequence [16] was cloned into the expression vector pMAL-c2e in frame with the 3′ end of the DNA encoding the maltose binding protein (MBP) to obtain pMAL-mAgo2 (Figure 1). In this vector, transcription is directed by the P_tac_ promoter and the encoded protein comprises the polypeptide chain of mAgo2 (859 amino acid residues) fused to the C-terminus of the MBP (388 amino acid residues). The choice of this vector followed several trials with other expression plasmids that failed to yield any soluble expression products.

**Figure 2.**
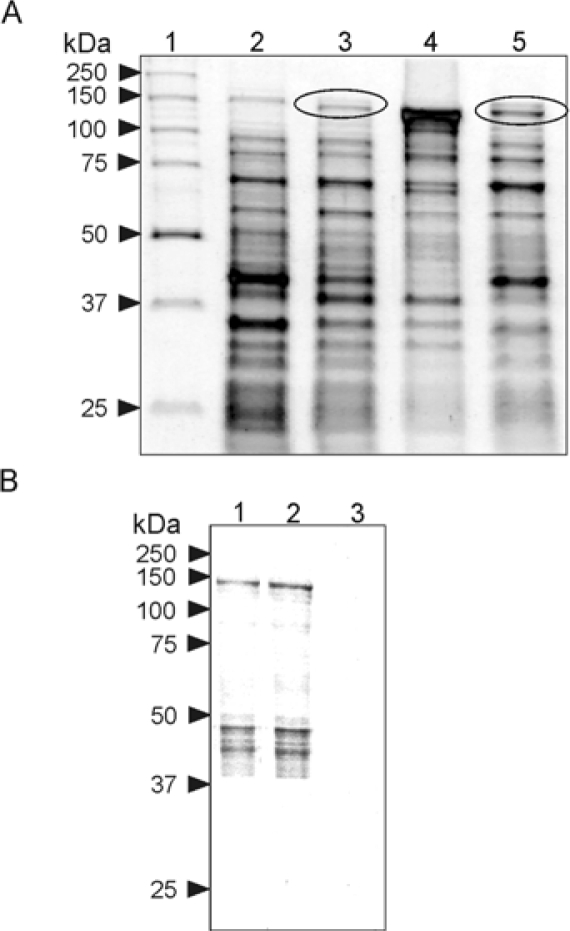
Expression and purification of MBP-mAgo2. Panel A: SDS-PAGE of lysates from uninduced (lane 2) and induced (lane 3) *E. coli* cells transformed with the expression vector pMAL-mAgo2; lanes 4 and 5 contain the insoluble and soluble phases of the bacterial lysate from induced cells, respectively; circles mark the expression product. Panel B: Protein sample eluted from the affinity chromatography on the amylose resin (lane 1); lanes 2 and 3 contain the soluble and insoluble phases of the eluate obtained by its centrifugation after two days of storage at 4 °C.

Plasmid pMAL-mAgo2 was then used to transform cells of the Rosetta™ *E. coli* strain. After induction with IPTG, the expression of MBP-mAgo2 fusion protein was assessed by SDS–PAGE analysis of the bacterial lysates (Figure 2A). The comparison of the protein patterns derived from induced and uninduced cells showed a band with the molecular size (~140 kDa) of the expected expression product (Figure 2A). Based on a densitometric analysis of the gel, the lysate derived from 0.5 L of bacterial culture contained ~3 mg of the fusion protein. The bacterial lysate from induced cells was then centrifuged and both the pellet and the supernatant were analyzed by SDS-PAGE to assess the solubility of the expression product. As shown in Figure 2A, a substantial amount of MBP-mAgo2 was found in the soluble phase of the bacterial lysate.

The bacterial extract was then subjected to an affinity chromatography with an amylose resin, using maltose for elution of bound proteins. SDS-PAGE analysis of the eluate revealed a substantial enrichment of the expression product that accounted for about 40% of the total proteins (Figure 2B). Its identity was then verified by the analysis the N-terminal amino acid sequence after blotting on a polyvinylidene fluoride membrane. Next, several attempts were made to improve the purity of the protein sample by (1) varying the conditions for the affinity chromatography and (2) introducing a gel-filtration step, but they were unsuccessful. It was also noticed that MBP-mAgo2 precipitated upon storage at −20 °C or −80 °C but it was stable at 4 °C for at least two days (Figure 2B), enough to perform the enzymatic assays.

### 2.2. Functional Characterization of Mouse Argonaute 2

**Figure 3.**
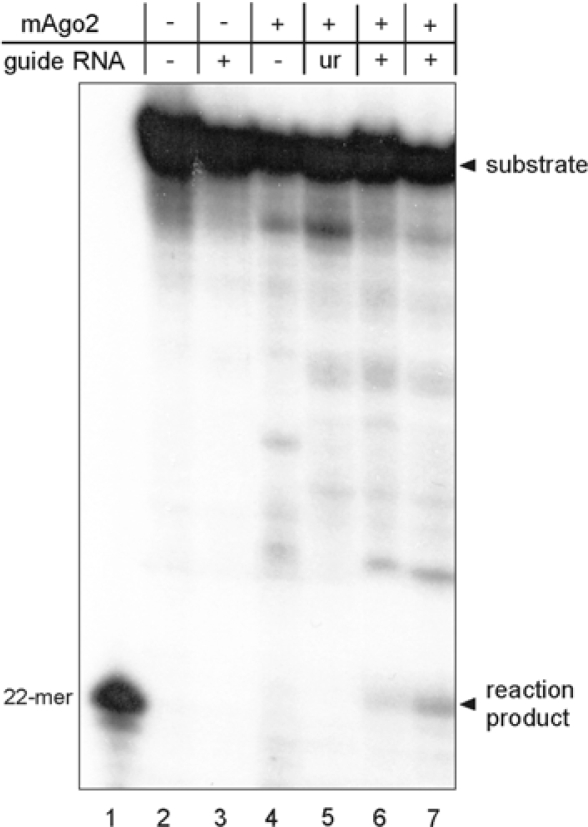
RISC assay for MBP-mAgo2. The enzyme was loaded with the guide siRNA and then incubated with a synthetic 50-mer RNA substrate that had been labeled with ^32^P at its 5′-end. Reaction products were revealed by denaturing PAGE followed by autoradiography. They included an unlabeled 28-mer product (not visible in the autoradiography) and a labeled 22-mer. Lane 1, molecular marker; lanes 2–7, reaction mixtures containing the labeled substrate, mAgo2 and/or the guide siRNA, as specified in the top of the figure; *ur* indicates a guide RNA with unrelated sequence, *i.e.*, not complementary to the substrate. Reaction mixtures contained 140 ng (lanes 4–6) or 280 ng (lane 7) of mAgo2.

Recombinant mAgo2 was then tested in a RISC assay, *i.e.*, for the ability to cleave a target RNA in a single position specified by a complementary siRNA [9]. For this purpose, mAgo2 was preincubated with a 5′-phosphorylated 21-mer guide oligoribonucleotide to form a minimal RISC. This complex was then mixed with a synthetic 50-mer RNA substrate that had been labeled with ^32^P at its 5′-end. The rationale for using this assay is that the binding of RISC to the substrate will produce a cleavage at a single position opposite to the phosphate between nucleotides 10 and 11 of the guide RNA. This will yield an unlabeled 28-mer product, and a labeled 22-mer corresponding to the 5′-fragment of the substrate, that can be revealed by denaturing PAGE followed by autoradiography. Obviously, this assay can be performed only if the enzyme sample is free of contaminating RNases that would otherwise degrade the substrate aspecifically. The electrophoretic analysis of the reaction products obtained with mAgo2 (Figure 3) revealed a band with the same mobility of a 22-mer marker, as expected for Argonaute 2 with a canonical enzymatic activity. No reaction product was detected when the assay was performed in the absence of mAgo2 or guide RNA. Finally, a reaction mixture containing mAgo2 and a guide RNA with unrelated sequence did not yield the 22-mer product (Figure 3). It should be noted that in these controls some aspecific degradation of the substrate also occurred, but none of the products comigrated with the Ago2-generated 22-mer product. Overall, these data indicate that mAgo2 expressed in *E. coli* can be loaded with a guide siRNA in absence of other cellular factor(s) and is able to cleave the target RNA at a single site that is dictated by the nucleotide sequence of the RNA cofactor, as described for human Ago2.

### 2.3. Mutational Analysis of mAgo2

**Figure 4.**
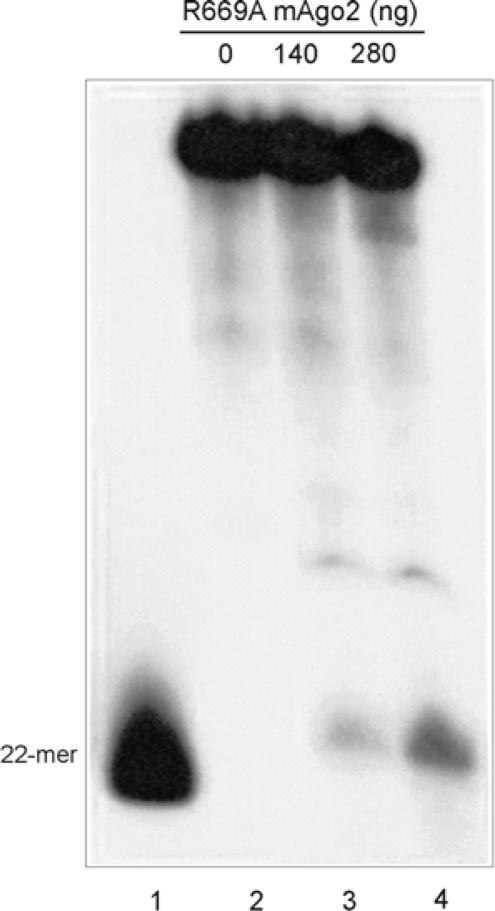
RISC assay for R669A mAgo2. Samples were treated as described in the legend of Figure 3. Lane 1, molecular marker; lanes 2–4, reaction mixtures containing the labeled substrate, the guide siRNA and the indicated amounts of R669A mAgo2.

It has been proposed that Argonaute 2 shares with RNase H a two-metal-ion catalysis mechanism [15]. It has also been proposed that Arg627 of *P. furiosus* Argonaute, equivalent to Arg669 of the murine protein, may be an active-site residue, possibly involved in substrate and/or B metal ion binding [15]. To test this hypothesis, we prepared a mutant mAgo2 with Arg669 replaced by an alanine (R669A mAgo2) and assayed it for RISC activity. As shown in Figure 4, a band corresponding to the 22-mer reaction product was clearly detectable, indicating that the R669A mAgo2 is enzymatically active. This result demonstrates that Arg669 of mAgo2 is dispensable for catalysis, and suggests that, if this residue really binds the metal ion B, an adjacent residue can functionally replace it. On the other hand, the results show that the bacterial expression system for mAgo2 is suitable for mutational studies.

## 3. Experimental Section

### 3.1. Materials and General Procedures

The expression vector pMAL-c2e and the amylose resin were obtained from New England Biolabs. SDS–PAGE analyses were performed according to Laemmli [17] using 10% (w/v) polyacrylamide gels that were stained with Coomassie blue. Plasmid pBS-mAgo2 [16], containing mAgo2 cDNA, was kindly provided by Dr. K. Saigo from the University of Tokyo. Oligonucleotides were purchased from Invitrogen.

### 3.2. DNA Cloning and Site-Directed Mutagenesis

The coding sequence of mAgo2 cDNA, was transferred in the expression vector pMAL-c2e by two cloning steps. In the first step, the 5′-end of the cDNA was obtained by PCR amplification of pBS-mAgo2 [16] with an upstream primer (5′-GGGGTACCGTACTCGGGAGCGGC-3′) and a downstream primer (5′-CCCAAGCTTCACTTGATGGATACCTTTAAGATG-3′) containing *Kpn* I and *Hind* III restriction sites (underlined), respectively. The amplification product was digested with *Kpn* I and *Hind* III and cloned into the same sites of pMAL-c2e to obtain pMAL-NmAgo2 (Figure 1). In the second step, the remaining part of coding sequence was excised from pBS-mAgo2 by cleavage with *Sma* I and *Hind* III and cloned in the same sites pMAL-NmAgo2 (Figure 1). The resulting expression vector, containing the entire coding sequence, was designated pMAL-mAgo2.

Site-directed mutagenesis was performed by replacement of a *Stu* I-*Stu* I wild-type segment of pMAL-mAgo2 (Figure 1) with a mutated DNA fragment obtained by PCR. The reaction was performed using an upstream primer (5′-CACCTGAAGAACACATACGCTG-3′) and a mutagenic downstream primer (5′-GCAGGCCTCTCTGATGGCCAGGAGCTCATGGTGGAGAACCTGCTGG AACTGGCCCTCGGAGACGCCATCG**GC**GTAG-3′; *Stu* I site is underlined and the mutated nucleotides are in marked in bold). The amplification product, containing the mutated site, was digested with *Stu* I and ligated into the vector to obtain pMAL-mAgo2 R669A. The structural identities of the wild-type and mutated expression vectors were verified by restriction analysis and DNA sequencing.

### 3.3. Protein Expression and Purification

The expression plasmid pMAL-mAgo2, containing the cDNA sequence coding for mAgo2, and the mutant plasmid pMAL-mAgo2 R669A were separately used to electroporate cells of the *E. coli* strain Rosetta™ (DE3) (Novagen). Transformed cells were then cultured at 37 °C in 0.5 l of LB broth containing 2 g/L glucose and 100 μg/mL ampicillin. When the absorbance of the culture at 600 nm reached a value of 0.5, cells were induced with 0.3 mM isopropyl β-d-thiogalactopyranoside (IPTG) at 25 °C for 4 h. Bacteria were then harvested by centrifugation at 4,000*g* for 10 min and washed with 250 mL of buffer A (50 mM Tris-Cl, 50 mM NaCl, 2 mM MgCl_2_, 10% glycerol, pH 7.4). Cells were resuspended in 100 mL of buffer A containing 4 mM DTT, disrupted by a French press operated at 1,800 psi, and centrifuged at 10,000*g* for 15 min at 4 °C. The resulting cell extract was then divided in 10 aliquots: one was processed for protein purification and the others were stored at −80 °C. The sample was mixed with 0.25 mL of amylose resin equilibrated with buffer A containing 4 mM DTT and incubated at room temperature on a rocking platform for 3 h. The resin was then washed twice with 3 mL of the same buffer and bound proteins were eluted with 0.3 mL of buffer A containing 4 mM DTT and 10 mM maltose. The eluate was then analyzed by SDS-PAGE and the expression product was quantitated by densitometric analysis of the gel. The final protein sample was stored at 4 °C and used for the enzymatic assays within 24–36 hours.

### 3.4. Structural and Functional Analyses

Protein sequence analyses were performed by automated Edman degradation of the polypeptide chain with an Applied Biosystems sequencer Procise™ model 491, equipped with a high-pressure liquid chromatography apparatus for identification of phenylthiohydantoin derivatives. The enzymatic activity of mAgo2 was tested by a gel-based assay, as described [14]. Briefly, a minimal RISC was assembled by incubating mAgo2 with a single-stranded 5′-phosphorylated siRNA (5′-UCGAAGUACUCAGCGUAAGdTdT-3′) in 20 μL of 50 mM Tris-Cl pH 7.4, containing 50 mM NaCl, 2 mM MgCl_2_, and 4 mM DTT, at 37 °C for 30 min. The sample was then supplemented with 1 μL of RNA substrate (5′-GAGGUGGACAUCACUUACGCUGAGUACUUCGAAAUGUCCGUUC GGUUGGC-3′), previously ^32^P-labeled at its 5′-end with T4 polynucleotide kinase and gel-purified. The resulting reaction mixture, containing ∼100,000 c.p.m., was incubated at 42 °C for 1 h and analyzed on a 6% polyacrylamide gel containing 8 M urea, followed by autoradiography.

## 4. Conclusions

Despite the great interest in Argonaute proteins and RNA interference, only one bacterial expression system for a eukaryotic Argonaute has been described so far [14]. It allows the expression of human Ago2 fused to Glutathione *S*-transferase (GST). However, the expression product was found to be poorly soluble and required coexpression of Heat shock protein 90 (HSP90) from a vector that is not commercially available. The present study shows that mouse Ago2 can be expressed as a fusion protein with the maltose binding protein in a soluble and enzymatically active form, without requiring coexpression with chaperones. We are aware that the final protein product is not sufficiently pure for structural studies. Nevertheless, the level of enzyme enrichment achieved with the affinity chromatography is sufficiently high to perform the RISC assay without suffering from the interference of contaminating RNases. This expression system is hence suitable for functional studies on Argonaute 2 and provides an additional tool to analyze the process of RNA interference. This may be particularly relevant for those studies that are performed on mouse models.

The results also contribute to the understanding of the catalytic mechanism of Ago2 by providing additional evidence that the enzyme can load the siRNA and cleave the complementary RNA in absence of other cellular factors. Finally it was found that mutation of Arg669, a residue previously proposed to be involved in substrate and/or B metal ion binding [15], to alanine doesn’t affect the enzymatic activity, suggesting that this residue doesn’t belong to the active site.
